# Nanowire dimer optical antenna brightens the surface defects of silicon

**DOI:** 10.1515/nanoph-2022-0742

**Published:** 2023-03-15

**Authors:** Ze Li, Qingzhang You, Hui Wang, Lisheng Zhang, Duan Zhang, Shangtong Jia, Yan Fang, Peijie Wang

**Affiliations:** The Beijing Key Laboratory for Nano-Photonics and Nano-Structure, Department of Physics, Capital Normal University, Beijing 100048, China; Key Laboratory of Semiconductor Photovoltaic Technology of Inner Mongolia Autonomous Region, School of Physical Science and Technology, Inner Mongolia University, Hohhot 010021, China; Elementary Educational College, Capital Normal University, Beijing 100048, China; State Key Laboratory for Mesoscopic Physics School of Physics, Peking University, Beijing 100871, China

**Keywords:** nanowire dimer (NWD), optical antennas, photoluminescence (PL) spectra, plasmonic gap modes, purcell effect, surface enhancement Raman scattering (SERS)

## Abstract

Plasmonic hot spots located between metallic dimer nanostructures have been utilized comprehensively to achieve efficient light emission. However, different from the enhancement occurred in the plasmonic hot spot, the investigation of light emission *off* the hot spot on submicron scale remains challenge. In this work, we have constructed a plasmonic nanowire dimer (NWD) system to brighten the light emission of the surface defects of silicon off the hot spot on the submicron scale. The NWD can trap light through plasmonic gap, then, the excited emitter on the submicron scale can radiate light efficiently by coupling with the dipole gap plasmonic mode. Furthermore, the coupling of dipole plasmonic mode with the emitters can be tuned by changing the gap size, and then photoluminescence emission was drastically enhanced up to 126 folds. Theoretical simulations reveal the photoluminescence enhancement arises from the combination of the NWD’s high radiation efficiency, Purcell enhancement, efficient redirection of the emitted photoluminescence and the excitation enhancement. In this study, the photoluminescence signal can be effectively enhanced by placing nano-antenna patch on the detected low-quantum-efficiency emitters, which may open up a pathway toward controlling plasmonic gap mode enhanced light emission off the hot spot on submicron scale.

## 1 Introduction

In microelectronics, silicon has been critical in the development of the electronics industry because of its many advantageous physical, electronic, and technological properties [1–3]. However, limited to the manufacturing process, the nanoscale surface roughness of the silicon wafer is unavoidable. This nanoscale surface roughness layer is a hotbed for the formation of surface defects of the nonbridging oxygens (NBOs, ≡ Si − O⋅, oxygen half-filled 2p orbital) [4–6], which photoluminescence (PL) band around 1.8 eV (680 nm), depending on its local environment [7–12]. But the photoluminescence (PL) intensity of these surface defects with limited lower concentration on silicon wafer is very weak, and these undesired surface defects seriously affect the lifetime and reliability of microchip systems. So the investigation of the surface defects of silicon is more problematic [13, 14].

The light emission of the emitter with low quantum yield can be increased by utilizing optical antennas such as plasmonic gap cavities [15, 16], which can tightly confine light in the vicinity of the optical emitters, enhancing the interaction between light and matter [7, 15–24]. Even the plasmonic resonators have proven to be a remarkably successful and robust platform for demonstrating a wide variety of optical phenomena. However, the two-dimensional (2D) flat emitters (Surface defects or transition metal dichalcogenides 2D optical materials) attached to silicon wafer are difficult to be integrated into the hot spot region of the plasmonic gap cavity [16, 22, 25, 28, 29]. In addition, once emitters excite lossy multipolar modes [30, 31], which lead to unacceptably high nonradiative decay and weakly coupled to the radiation field, thus limiting the achievable coupling strength. In this study, our motivation was to boost inconspicuous light emission off the hot spot using a low lossy plasmonic bonding dipole mode supported nanowire dimer (NWD) optical antenna [26]. This provides a new avenue to realizing the enhancement of light emission of emitters located off the hot spot at a submicron ( > 100 nm) distance.

## 2 Results and discussion

**Figure 1:**
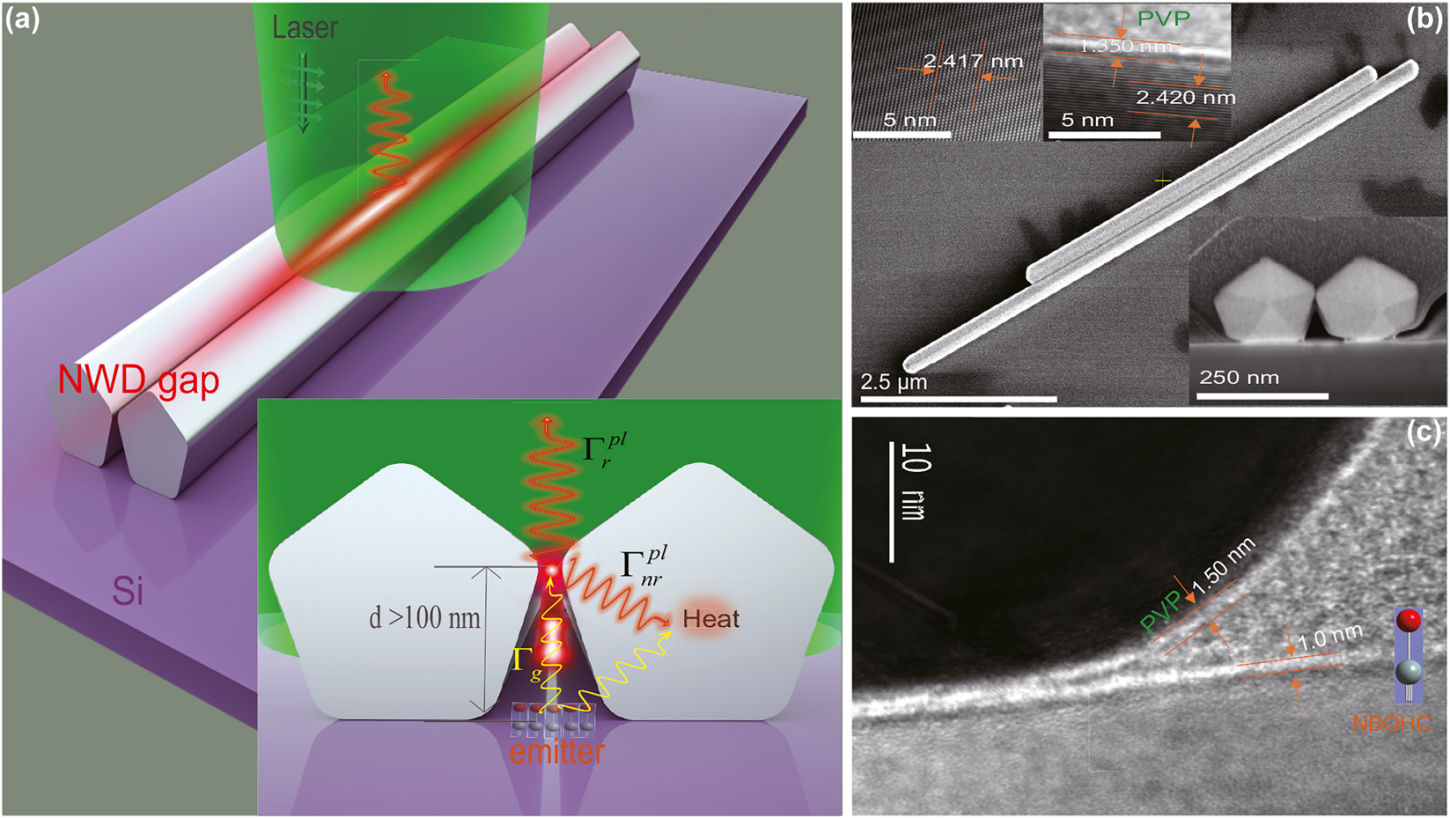
Schematic diagram and sample characterization. (a) Gap plasmon nanocavity formed by nanowire dimer (NWD) setup. The cavity is composed of NWD separated by a gap between their edges. Light emission from the emitters positioned beneath the NWD on submicron scale can be enhanced by the NWD optical antenna. Left inset is the cross section diagram showing the coupling process of the gap plasmon with the emission light from the surface defect *off* the hot spot on submicron scale (*d* > 100 nm). (b) SEM images of a typical Ag NWD. The inset of lower right is the SEM cross-sectional image of a NWD system, and the gap is around 5 nm; the upper left insets are high-resolution TEM cross-sectional images of crystal structure, showing the crystallinity and corresponding growth directions that are characteristics of silver nanowires synthesized using the present method. Single-crystal lattice distance of the Ag facet near the gap is about 0.24 nm, which corresponds to the Ag[111] lattice spacing. The polyvinylpyrrolidone (PVP) molecules layer enveloped the Ag nanowire is about 1.35 nm in thickness. (c) High-resolution TEM cross-sectional image of the nanowire with the silicon substrate structure, showing the PVP layer thickness and the surface roughness layer. The silicon surface roughness is about 1 nm. Here, the surface defects formed by non-bridging oxygens (NBO) positioned in this silicon surface roughness layer as shown in lower right inset.

Gap plasmon nanocavity setup is illustrated in Figure 1(a). The pentagon edges of nanowires dimer (NWD) are closely adjacent, which increases the lightning rod effect [32]. Figure 1(a) demonstrates that the exciting green light can be converged to the nanogap between two pentagon edges and transmit to the submicron scale region where emitter is located. Meanwhile, the emitter beneath the NWD can be excited, its spontaneous emission will radiate to the far field via coupling to the NWD nanoantenna. Once plasmon gap mode in NWD are excited by the emitter through the coupling, it can radiate light into free space, at radiative rate $\Gamma_{r}^{pl}$ , or can be lost through Ohmic losses, at nonradiative rate $\Gamma_{nr}^{pl}$ . The relative values of these radiative and nonradiative rates depend on the size and composition of the NWD. All of the processes involved in modified emission by NWD are illustrated schematically in Figure 1(a) left inset (indicated by the red arrows). The rate at which light is emitted to the far field is as the follows formula [33]:

(1) $$\Gamma_{\text{far}}=\Gamma_{0}+\Gamma_{g}\;*\;\Gamma_{r}^{pl}/\left(\Gamma_{r}^{pl}+\Gamma_{nr}^{pl}\right)$$

here, Γ_0_ represents the rate at which the emitter radiates directly into free space, without coupling to the NWD. The term Γ_*g*_ represents the radiative rate of an emitter coupling to the NWD (see SI part I). However, Γ_0_ ≪ Γ_*g*_, so Γ_0_ has little overall effect on the far field emission rate [34]. And the term of $\Gamma_{r}^{pl}/\left(\Gamma_{r}^{pl}+\Gamma_{nr}^{pl}\right)$ in Eq. (1) is the antenna’s radiation efficiency (*η*), that usually have a maximum value for the dipole mode and a minimum value lossy multipolar mode.

We started with the experimental morphology characterization. Figure 1(b) shows this NWD nanostructure formed by two 190 nm diameter Ag nanowires with the gap distance approximately 5 nm. A high-resolution TEM image of Ag nanowires is shown in the right inset of the Figure 1(b), which confirms that the Ag nanowire structure is single crystalline, and their facets and edges are smoothed at the atomic level. This high-quality nanowire guarantees less nonradiative decay [34–43]. The polyvinylpyrrolidone (PVP) layer outside the nanowire (Figure 1(b) and (c)) was used as the spacer between the plasmonic resonator and bulk silicon. Figure 1(c) (and SI, Figure S1) shows the cross section TEM image of the silicon surface roughness layer which is approximately 1 nm. This nanoscale surface roughness layer is critical for the formation of surface defects of the nonbridging oxygens (NBO) [4–6], which photoluminescence (PL) band around 1.8 eV varying with its local environment [7], [8], [9], [10], [11], [12, 44, 45]. Owing to the small amount of oxygen element, it is difficult to observe the related characteristic spectrum. However, the low quantum efficiency NBO emitter on the silicon surface is an ideal prober for this research. Next, we deposited the silver nanowire dimers on the surface of silicon wafer to perform the PL experiments.

**Figure 2:**
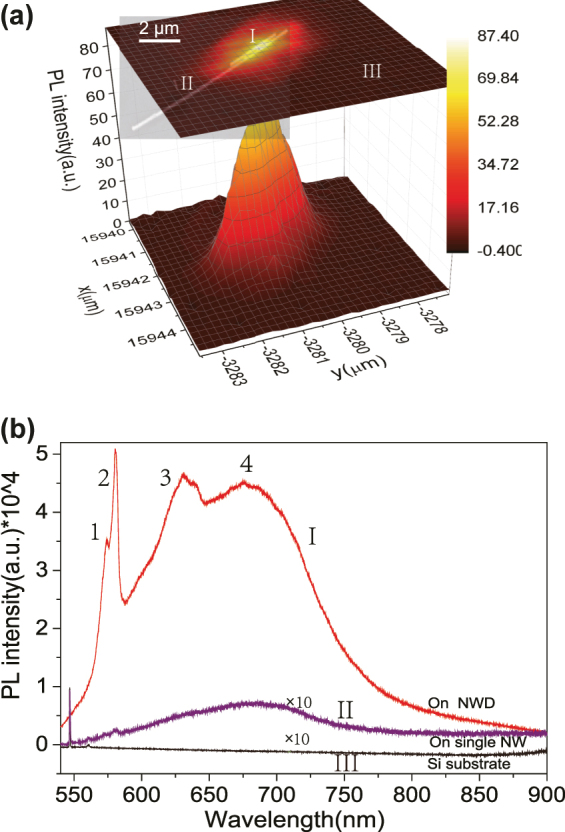
Experimental demonstrations and photoluminescence (PL) spectral enhancement of the surface defect coupled to the nanowire dimer. (a) The 2D and 3D image mapping of PL with the 680 nm peak of the surface defect of silicon on the Ag NWD system. (b) The room temperature photoluminescence (PL) spectra of the Ag NWD on silicon substrate system, here spectra of I, II, and III correspond to the positions shown in (a), which are on the NWD, single nanowire, silicon substrate, respectively.

Figure 2(a) shows the 2D and 3D image mapping of the NWD system with the PL peak at 680 nm. The SEM image of the NWD is shown in Figure 2(a), which overlap with the corresponding position of the 2D image sample mapping with the PL peak at 680 nm. Here, the polarization direction of the exciting light is perpendicular to the NWD’s axis in order to excite the plasmonic gap mode (PGM). Furthermore, there is no enhancement if the polarization of exciting light is along the nanowire axis (see SI Part VI. “THE POLARIZATION DEPENDENCE OF PL WITH NWD”). For the single nanowire part (Positon II shown in Figure 2(a) and the purple line II in Figure 2(b)), it’s PL enhancement is negligible, and the PL mapping shown in Figure 2(a) also confirmed the PL intensity on the single nanowire part was similar to the PL intensity of background silicon substrate (Positon III shown in Figure 2(a) and the black line III in Figure 2(b)). A maximum PL intensity distribution just located at the position of the NWD gap does appear (Positon I shown in Figure 2(a) and the red line I in Figure 2(b)). Furthermore, if we remove the background signal of PL mapping covering the NWD, then only the PL intensity on the NWD gap remains (see SI part II “CONFIRMATION THE PL BANDS ENHANCED BY NWD”). Strong PL enhancement up to 62 folds by comparing the PL spectra on the NWD gap (I in Figure 2(b)) with that on the silicon substrate off the NWD (III in Figure 2(b)).

**Figure 3:**
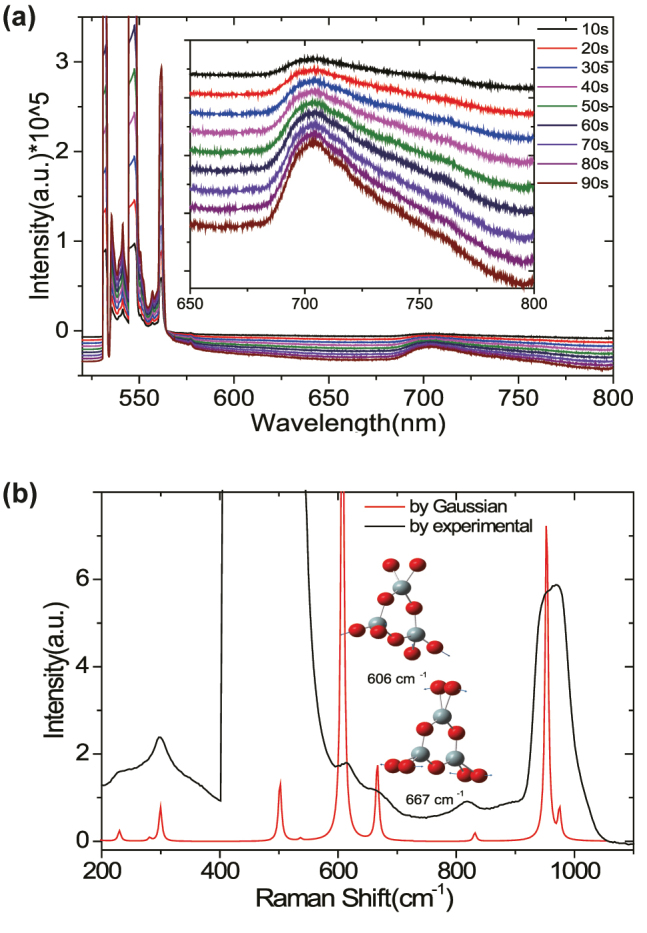
The PL and Raman spectra of the silicon substrate. (a) The PL spectra of the silicon substrate background by 532 nm excitation with the higher laser power (18 mW) and via longer accumulating time 20 s, 30 s, …, 100 s. Inset is the amplification of the wavelength range from 630 nm to 800 nm. (b) The black line is the experimental Raman spectroscopy of silicon by 532 nm excitation. The red line is the theoretical result of three-membered ring Raman scattering spectroscopy via Gaussian simulation. The inset shows the typical Raman modes at 606 cm^−1^ and 667 cm^−1^ of three-membered ring.

The main pronounced PL peak 4 at 680 nm in Figure 2(b) is carefully attributed to the surface defects (the nonbridging oxygens, NBOs, ≡ Si − O⋅) of silicon substrate, which is correspoding to the weak PL peak of 700 nm in the Figure 3(a). The blueshift of the PL peak is due to the coupling of surface defects with plasmonic NWD. The surface defects were formed by nonbridging oxygens positioned in this silicon surface roughness layer. Owing to the small amount of element oxygens, it is difficult to observe the related characteristic spectrum. Hence, the PL spectra of the silicon substrate itself (No NWD present) with higher laser power (18 mW) and longer accumulating times were performed as shown in Figure 3(a). There are several peaks occurred near Rayleigh line (by 532 nm excitation), which are attributed to the Raman scattering from the surface defect of silicon as reported in the ref. [44]. The strained three-membered rings are considered as the precursor of oxygen dangling bonds, and its Raman mode at 606 cm^−1^ can be used for evaluating the amount of oxygen dangling bonds [6]. Figure 3(b) shows the theoretical calculations Raman spectra of the three-membered silicon oxygen ring, which were performed with the Gaussian 09 software using density functional theory, the B3LYP functional 32 and 6–31G(d) basis set. The typical Raman modes at 606 cm^−1^, 667 cm^−1^ are Si − O symmetric rocking, Si − O anti-symmetric rocking, respectively. They are consistent with our experimental Raman spectra (Figure 3(b) black line), which is the amplification part of the spectra shown in Figure 3(a). So the weak PL bands at 700 nm (Figure 3(a) inset) were assigned to the light emission from surface nonbridging oxygens (NBOs). For the 520 cm^−1^ and 980 cm^−1^ peaks in Figure 3(b) are well known as the first order and second order Raman peaks of silicon. The 520 cm^−1^ peak is saturated due to long time accumulating in case to observe the weak Raman modes of surface defects of silicon.

The observed PL spectrum in Figure 3(b) also shows a fine structure (see SI part III “Proof of Raman Peaks by Different Exciting Wavelengths”, Figure S3), the peaks of 1, 2, and 3 at 573 nm, 582 nm, and 631 nm were attributed to the surface-enhanced Raman scattering (SERS) of the PVP molecules (which is enveloping the nanowires near the gap region, see (Figure 1(b) and (c)). The strong enhancement of SERS peaks of 1, 2, and 3 confirm the highly confined near-field in the gap cavity of NWD (see SI part IV(C), Figure S5(c)).

**Figure 4:**
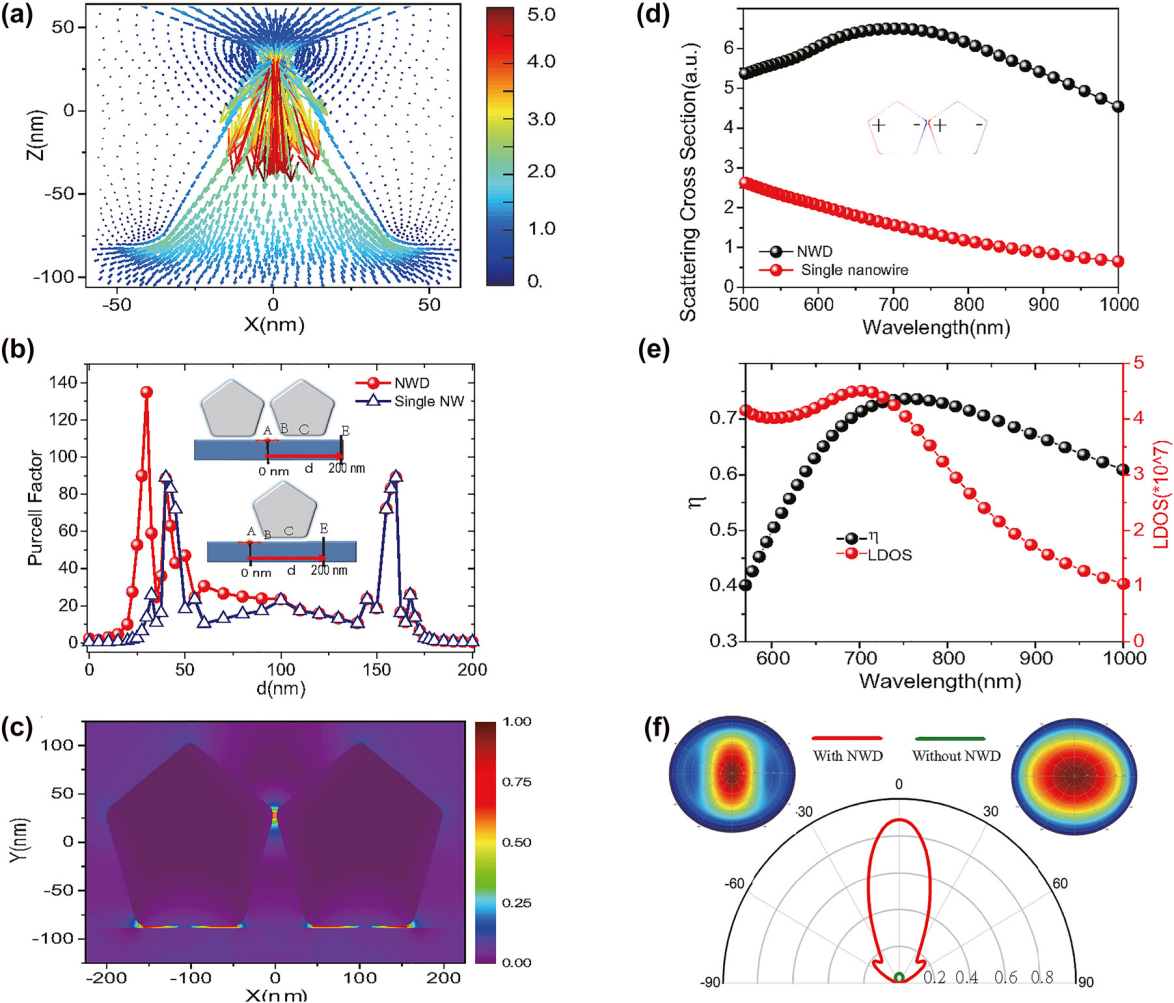
Theoretical investigation of plasmonic gap mode modifies the efficiency of convergence and excitation. (a) The calculated Poynting vectors distribution in the NWD gap region by 532 nm excitation. (b) Purcell factor *F*_*P*_ of the emitter varying with distance *d* as shown inset (point A, *d* = 0 nm; point C, *d* = 100 nm; and point E, *d* = 200 nm). (c) The NWD’s electric-field distribution excited by a dipole (*λ* = 680 nm) at position A. (d) Scattering spectrum of NWD. Inset is the calculated charge distribution at the resonance peak. (e) The LDOS, and radiation efficiency *η* of the Ag NWD (here, we set the single NW diameter is at 190 nm, and the gap of DNW is 5 nm). (f) Radiation patterns (this shows the part upward to air) of a dipole emitter placed on planar silicon substrate. Left and right insets present the far-field projection in a plane above the interface of the silicon substrate with and without NWD.

These experimental phenomena in Figure 2 bring several critical questions. Why single nanowire cannot effectively enhance the PL signal of NBOs on the silicon surface. However, once this single nanowire coupled to another nanowire to form the nanowire dimer (NWD) system, the giant PL enhancement of the NBO on the silicon surface would occurs. What’s the enhancement mechanism of the PL of NBO emitter located off the gap hot spot at submicrometer? Next, these interesting questions were investigated by the finite-difference time-domain (FDTD) simulation [46] (see SI part IV(A)). Shown in Figure 4(a) is the calculated Poynting vectors, which demonstrated the exciting light (532 nm) was converged to the NWD gap and then radiated downward to surface of silicon substrate. The NWD can excite a larger area of emitters (the region between the corners of two nanowires with silicon substrate at submicrometer, see SI part IV(B), Figure S5) by comparing with the single nanowire. Once an emitter was stimulated, one should consider the enhancement of the quantum yield (*q*/*q*_0_) for the emitter in the total enclosed cavity including the bottom edges of the NWD with the silicon substrate interface. Due to the limit of a low quantum yield for the emitter, i.e. the nonradiative decay $\gamma_{nr}\;\gg \;\gamma_{r}^{0}$ (the radiative decay), so the enhancement of the quantum yield for an emitter can be written as *q*/*q*_0_ = *F*_*P*_(*γ*_*r*_ + *γ*_*nr*_)/(*F*_*P*_ *γ*_*r*_ + *γ*_*nr*_) ≈ *F*_*P*_ [47]. The Purcell factor at a wavelength *λ*_*em*_ can be defined as: *F*_*P*_ = *γ*_*r*_/ $\gamma_{r}^{0}$ , where *γ*_*r*_ and $\gamma_{r}^{0}$ are the rates of emitter modified by the NWD and placed on the planar silicon, respectively. The Purcell factor is dependent on distance *d*, which is shown in Figure 4(b). When the dipole emitter is positioned near point B (*d* = 40 nm) as shown in 4b inset of NWD case, the Purcell factor is significantly increased comparing with that value as the dipole emitter at A point (*d* = 0 nm). So the dipole emitter in such total enclosed NWD cavity will experience larger quantum yield enhancement by comparing with the Purcell factor of the single nanowire. Meanwhile, Figure 4(c) shows the NWD’s electric-field distribution excited by a dipole (*λ* = 680 nm) at position A. The plasmonic gap mode is excited by the dipole either, then it radiate light into far field at radiative rate $\Gamma_{r}^{pl}$ by coupling with the NWD gap cavity. According to Eq. (1) we would optimize the NWD’s radiation efficiency (*η*) to improve the total light emission next.

Figure 4(d) shows the calculated scattering spectrum of NWD. The pronounced scattering peak of plasmonic gap mode (PGM) located at approximate 715 nm. It was identified to be the bonding dipole mode by analyzing the induced charge distribution (Figure 4(d) inset). This bonding dipolar mode is regarded as the super-radiative mode owing to the large size of the total electric dipole moment, which is proportional to the diameter of the nanowire [48].

The calculated radiation efficiency *η* and the LDOS [49–52] are demonstrated in Figure 4(e), both peaks of *η* and LDOS are aligned at 715 nm. And the LDOS feature closely resembles the scattering spectra shown in Figure 4(d). These merit properties insure the dipole mode supported NWD have an excellent capability to radiate the light with 715 nm [35]. However, for single nanowire part, the higher-order mode with low radiation efficiency will be excited, (see SI part IV(C), Figure S5(e) and (f)), and this limits the light emission of emitter, so this is the reason why we can’t observed the PL enhancement in the single nanowire part (Figure 2(b)) experimentaly.

Figure 4(f) shows the collection efficiency which is considerably increased by the presence of the NWD nano-antenna. Firstly, only a dipole (*λ* = 680 nm) polarized along the *x*-direction on the silicon substrate was considered, the two-dimensional radiation intensity patterns are shown in Figure 4(f) green line (which only shows the part upward to air). Secondly, a NWD optical antenna is introduced and a completely different angular emission pattern appears, as shown in Figure 4(f) red line. The maximum of the radiation intensity increase by one order of magnitude compared to the case without the NWD antenna. The light radiates more efficient via localizing to the narrow *slit-like region* from the far-field projection in the plane above the silicon substrate (see Figure 4(f) left inset) compared with that without NWD (see Figure 4(f) right inset). Here, the emitted radiation is coupled to a detector using collection through numerical aperture NA = 0.7. So, we achieved a *η*_collection_ = 15 times collection efficiency.

To summarize the light emission enhancement, our theoretical simulations reveal the PL enhancement arises from the combination effects of the NWD’s high radiation efficiency, Purcell enhancement, efficient redirection of the emitted PL and the excitation enhancement. In order to compare with experimental results, we took into account the above four effects and introduce an effective enhancement factor *EF*_eff_ defined as the product of four factors [47]:

(2) $$EF_{\text{eff}}=\eta \;*\;\eta_{\text{collection}}\;*\;E_{\text{exc}}/E^{0}\;*\;\gamma_{r}/\gamma_{r}^{0}$$

here, the term *E*_exc_/*E*^0^ describes the ratio of the excitation enhancement (see SI part IV(D), Figure S6(a)). We integrated the effective enhancement factor overlap the position of the emitter from *d* = 0 nm to *d* > 200 nm and the averaged enhancement factor value *EF*_eff_ is 190 (see SI part IV(D)), which is fairly compared with the experimental result.

**Figure 5:**
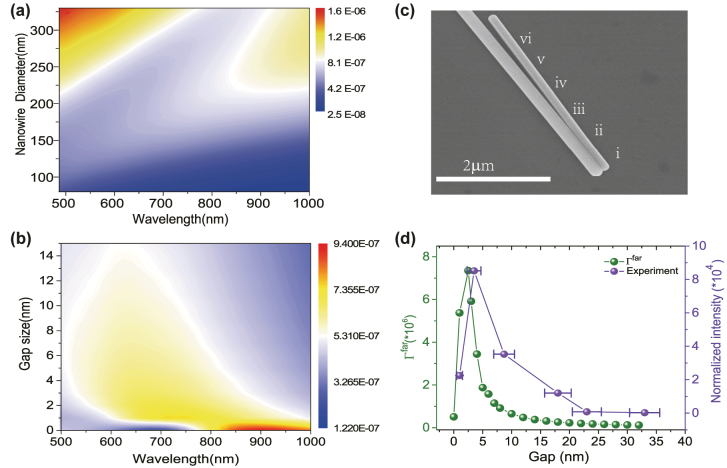
Unparallel NWD optical antenna. (a) The scattering spectrum of the Ag NWD as it evolves with the single nanowire diameters. (b) The scattering spectrum of the Ag NWD as it evolves with the gap size. (c) The SEM image of unparallel Ag NWD. (d) Far-field emission rate Γ^far^ compared with the PL intensity of 680 nm collected at points i, through vi corresponding to the SEM image positons in (c).

It’s noted that the resonance peak of *gap dipole mode* can be tuned by changing the nanowire diameters and the gap size between NWD. Figure 5(a) shows the calculated scattering spectrum of the Ag NWD evolves with nanowire diameters (the gap is fixed at 5 nm). When the diameter of nanowire is 165 nm, the corresponding plasmonic resonance peak will match the NBO’S PL peak at 690 nm. Based on this simulation, we prepared nanowires with diameter about 165 nm experimentally. And our experimental results confirmed most plasmonic resonance peaks of NWDs are nearby 700 nm (see SI part V, “The Dark field Scattering Spectrum Of NWDs”, Figure S10). Meanwhile, the effects of variation gap size of NWD were explored as shown in Figure 5(b). Then, in order to achieve stronger enhancements of light emission, an unparallel NWD system was designed to change the gap size of NWD as shown in Figure 5(c) here, an unparallel NWD was formed by two closely adjacent nanowires differing in diameter (one nanowire with a diameter of 166 nm and another with a diameter of 253 nm). The gap size changes continuously from 0 nm at the contact end (point i) to 33 nm (point vi) to the separate end. Figure 5(d) shows the PL intensity at 680 nm of points i to vi. With the gap size increasing, for the region of point ii (about 2.5 nm–3.5 nm), we obtained a giant PL enhancement up to 126 folds.

Because the Purcell factor *F*_*P*_ of the emitter located off the hot spot on submicron distance (115 nm) is insensitive to the change of the gap size, and also both the collection efficiency and the excitation enhancement only have a slight change. Therefore, we only need to consider the dependence of radiation efficiency and LDOS on gap size (at *λ*_*em*_ = 680 nm), the calculated far-field emission rate Γ^far^ ≈ η*LDOS (see Figure 5(d)) almost coincide with the experimental results. When the gap size is about 2.5 nm ∼ 3.5 nm, we get a maximum enhancement. When gap size increases to a certain size, such as points vii and viii (approximate 22 nm ∼ 32 nm), the PL signals become very weak, indicating these two nanowires no longer coupling. This analysis demonstrated that the light emission enhancement off the hot spot on submicron scale can be tuned by changing the gap size of the NWD.

## 3 Conclusions

In this study, we designed the Ag NWD optical antenna systems to enhance the sponetaneous emission of surface defects of silicon beyond the hot spot. It is different from common emission enhancement *in* the plasmonic hot spots; the investigation of light emission here is focused on the emitter which is *off* plasmonic hot spot on the submicron scale. It was demonstrated that the NWD optical antenna can converge the incoming light and then the upper and lower anti-symmetry constructed of the gap region insure more light transmit to a submicron far-field distance that the emitters located. The surface emitter’s spontaneous emission was greatly modified by the NWD gap cavity. The light emission to the far field was more efficient as it improved of the collection efficiency and the coupling to high quality and low Ohmic losses bonding dipole mode supported by NWD. Furthermore, we proposed this brand new physical approach is expected to be universal for 2D form materials. Overall this research proves the PL signal can be effectively enhanced by placing the nano antenna patch on the detected low-quantum-efficiency emitters, and also open up a pathway toward controlling PGM-enhanced spontaneous emission beyond the hot spot on the submicron scale.

## 4 Experimental details

*Synthesis of silver nanowire*: The silver NW was synthesized via wet-chemistry polyol reduction method [27], 10 mL of ethylene glycol was placed in a glass beaker on a magnetic stirrer, and then 700 mg of polyvinylpyrrolidone (PVP) and 1000 mg of AgNO_3_ were slowly added and stirred until fully dissolved. Next, the mixed solution was placed in a sealed Teflon reactor, which was heated at 160 °C for 90 min. The reactor was then allowed to cool, and the product was washed with acetone and alcohol, centrifuged (2000 rpm, 10 min) and redispersed in ethyl alcohol (10 mL). Finally, a highly pure solution of silver NWs was obtained. The high-quality silver NW was ∼10 ± 5 μm in length and ∼200 ± 30 nm in diameter from scanning electron microscope (SEM). The chemically synthesized Ag nanowires are well prepared to guarantee the less nonradiative decay which is nearly free of any defects, grain boundaries and surface roughness to form a high-quality nanogap antenna. The Ag NW is covered by a PVP layer used as the spacer. Its thickness can be checked by the high resolution TEM image of typical Ag NW surfaces. The sample was prepared as the follows: The Ag NW solutions were drop-cast on a copper grid, and then dried by nitrogen gas. The PVP with an average thickness about 1.5 nm after the washing process, was obtained by averaging from five nanowires.

*Characterization*: The photoluminescence (PL) spectra were recorded using a RH13325 (R-2000) spectrophotometer. The samples were excited with 532 nm wavelength lasers. The samples were excited with 532 nm wavelength lasers and the polarization of the exciting laser was perpendicular to the long axis direction of the NWD. A 50× objective lens was used to achieve a 180° backward scattering configuration. The diameter of laser spot at sample is approximately 1 μm. SEM images of the NW were obtained using a HitachiS-4800 microscope. High-resolution TEM images for dimer nanowire were taken with a FEI2.0 TEM microscope, TEM images were taken at an accelerating voltage of 200 kV and magnification of 50,000×.

## 5 Electronic supplementary material

Supplementary material is available from the author. Contents include “Quantum Electrodynamics (QED) Treatment; Confirmation the PL Bands Enhanced by NWD; Proof of Raman Peaks by Different Exciting Wavelengths; Theoretical Simulation Analysis; The Dark field Scattering Spectrum of NWDs; The Polarization Dependence of PL with NWD.”

## Supplementary Material

Supplementary Material Details
