# Supplementary material for: Nanowire dimer optical antenna brightens the surface defects of silicon
Source: Nanophotonics. 2023 Mar 15;12(9):1723–31. doi: 10.1515/nanoph-2022-0742 (PMC11501950; doi:10.1515/nanoph-2022-0742)
Supplement: Supplementary file 1 — Supplementary Material Details [file j_nanoph-2022-0742_suppl.pdf]

Supporting Information for "Nanowire Dimer Optical Antenna Brightens the Surface Defects of Silicon"

Ze Li[1,2†], Qingzhang You[1†], Hui Wang[1], Lisheng Zhang[1], Duan Zhang[1,3], Shangtong Jia[4], Yan Fang[1], Peijie Wang[1*]

[1] *The Beijing Key Laboratory for Nano-Photonics and Nano-Structure, Department of Physics, Capital Normal University, Beijing 100048, China;*

[2] *Key Laboratory of Semiconductor Photovoltaic Technology of Inner Mongolia Autonomous Region, School of Physical Science and Technology, Inner Mongolia University, Hohhot 010021, China;*

3 *Elementary Educational College, Capital Normal University, Beijing 100048, China;*

4 *State Key Laboratory for Mesoscopic Physics School of Physics, Peking University, Beijing 100871, China.*

[†] *Co-first authors.*

(Dated: February 19, 2023)

PACS numbers:

I. QUANTUM ELECTRODYNAMICS(QED)TREATMENT

The treatment of spontaneous emission requires the theoretical treatment of quantum electrodynamics(QED). It was found that the light emission mainly related to the local density of state(LDOS) of the nanoantenna[1–3]. In general, the spontaneous emission rate of a dipole in free space is given by

$$\Gamma_0 = \frac{\omega_o^3 \mu_{12}^2}{3\pi\varepsilon_o \hbar c^3} \quad (1)$$

where $\hbar\omega_0$ is the energy difference between the initial and final electronic states and μ_{12} is the dipole matrix element between these two states. This rate is the inverse of the lifetime of the excited state of the emitter. For an emitter that not in free space (in the plasmonic gap or in the vicinity of mental nanoparticals) gives a modified emission rate:

$$\Gamma_g = \frac{2\pi \mu_{12}^2 E_o^2}{\hbar^2} \rho(r, \omega_o) \quad (2)$$

$\rho(r, \omega_o)$ is the LDOS at frequency ω_o at position r. That is, $\rho(r, \omega_o)$ is the number of electromagnetic modes, per unit frequency and per unit volume. Generally, the LDOS describes the available optical eigenmodes in which photons can exist at a specific spatial location, and the optical eigenmodes occurring in an antenna will be encoded in the measured plasmon resonances signal. Our simulation approach essentially relies on the LDOS which can be expressed in terms of the Green's function tensor. The Green's function tensor accounts for the energy dissipation of a dipole in an arbitrary inhomogeneous environment [4, 5].

In general, the spontaneous emission rate of a dipolar emitter in a the nanowire dimer gap can express the photoluminescence emission processes from the silver nanowire [1].

$$\rho_{\mathbf{P}}(\mathbf{r}_o, \omega) = \frac{6\omega}{\pi c^2}[n_P \cdot Im\{\vec{G}(\mathbf{r}_o, \mathbf{r}_o, \omega)\} \cdot n_P] \quad (3)$$

The Green's function used in Eq.(3) is indirectly defined by the electric field $\mathbf{E}$ at the observation point $\mathbf{r}$ generated by a dipole $\mathbf{p}$ located at $\mathbf{r}_o$.

$$\mathbf{E}(\mathbf{r}) = \frac{1}{\varepsilon_0}\frac{\omega^2}{c^2}\vec{G}(\mathbf{r}_o, \mathbf{r}_o, \omega)\mathbf{p} \quad (4)$$

According to Poynting's theorem the power dissipated by a time-harmonic system is

$$\mathbf{P} = \frac{\pi\omega^2}{12\varepsilon_0}|\mathbf{p}|^2 \rho_{\mathbf{P}}(\mathbf{r}_o, \omega) \quad (5)$$

This known expression denotes the power emitted by a classical dipole radiation in free space

$$\mathbf{P}^o = |\mathbf{p}|^2\omega^4/(12\pi\varepsilon_0 c^3) \quad (6)$$

we can express the LDOS in terms of the normalized power radiation

$$\rho_{\mathbf{P}}(\mathbf{r}_o, \omega) = \frac{\omega^2}{\pi^2 c^3}\mathbf{P}/\mathbf{P}_o \quad (7)$$

The factor $\frac{\omega^2}{\pi^2 c^3}$ corresponds to the LDOS in free space and is used in the standard derivation of blackbody radiation. The power $\mathbf{P}$ in Eq.(5) accounts for the total dissipated power, usually, plasmon resonances in nanostructures can be damped radiatively by re-emission of a photon or non-radiatively through the creation of hot electronChole pairs via Landau damping, so $\mathbf{P}$ is the

*Electronic address: pjwang@cnu.edu.cn

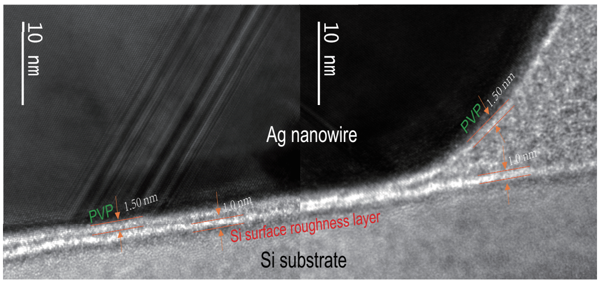

Fig. S 1: High-resolution TEM cross-sectional image of the nanowire with the silicon substrate structure, showing the PVP thickness and the surface roughness layer. The surface roughness is about 1 nm. Here, the surface defects formed the nonbridging oxygen hole centers(NBOHCs) positioned in this silicon surface roughness layer.

sum of radiated power$\mathbf{P}_{rad}$ and power dissipated into heat (ohmic loss) and other channels ($\mathbf{P}_{loss}$). So, the branching ratio between these two decay mechanisms is the antenna radiation efficiency $\mathbf{E}_{rad}$, it is a useful figure of merit to describe the light emission of the antenna:

$$\varepsilon_{rad} = \frac{\mathbf{P}_{rad}}{\mathbf{P}} = \frac{\mathbf{P}_{rad}}{\mathbf{P}_{rad} + \mathbf{P}_{loss}} \tag{8}$$

While $\mathbf{P}$ is most conveniently determined by calculating the field $\mathbf{E}$ at the dipole's position according to the following formula:

$$\mathbf{P} = \frac{\omega}{2} Im\mathbf{p}^* \cdot \mathbf{E}(\mathbf{r}_o) \tag{9}$$

II. CONFIRMATION THE PL BANDS ENHANCED BY NWD

The experiment of room-temperature PL mapping were carried out using a 532 nm excitation laser with 0.78 mW incident power. The SEM images of the nanowires dimer(NWD) was shown in Fig.S2a, here both nanowires with a diameter of about 195 nm. Then we performed the PL mapping on the gap of NWD as shown in Fig.S2b at the wavelength 680 nm which is attributed to the e-mission signal of surface defect of silicon substrate. It presents a maximal distribution in the plasmonic gap region between the double nanowires. Furthermore, if we removed the background signal of PL mapping covering the NWD, only the PL of silicon in the gap was left as in Fig.S2c. This confirmed that the enhancement of PL of silicon just results from the gap of NWD.

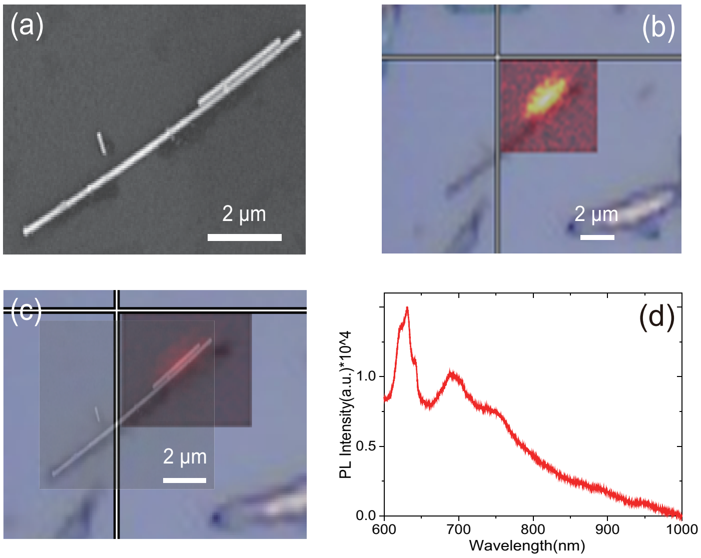

Fig. S 2: (a), The SEM images of the nanowires dimer(NWD), here the single nanowire with a diameter of about 195 nm; (b), The PL mapping with the 680 nm peak of silicon for the region covering the NWD and overlapping the image under the view of optical microscopy; (c), The overlapping the SEM image of NWD in (a) to (b) again by removing the background signal of PL mapping covering the NWD as in (b); (d), The typical PL spectra in the gap of the NWD region.

III. PROOF OF RAMAN PEAKS BY DIFFERENT EXCITING WAVELENGTHS

Comparison of the PL spectra of bulk silicon chip under different exciting laser wavelength of 532 nm and 633 nm were demonstrated in Fig.S3 (a). Here we attribute the PL band 4 (700 nm) to be the PL of the surface defect of silicon. However, the peaks of 1, 2 and $1'$,$2'$ in the PL spectra (Fig.S3a)should be attributed to the SERS(Surface Enhanced Raman scattering) of $C = C$ of PVP molecules. In fact, the attribution of peaks 1, 2, 3 in the PL spectra was a long controversial problem in Nature Photonics by Cho et al[8]. This letter by Cho et al was commented by Russell et al[9–11].

It should be noted that the PL band position does not change with different excitation wavelengths. However, these SERS band positions in the PL spectrum change with different excitation wavelengths with fixed Raman shifts from the Rayleigh line. So we again attributed these peaks as the SERS of of $C = C$ of PVP molecule as the followings: We transformed the x-axis of the PL spectra wavelength (nm) to be the Raman shifts (cm^{-1}) via referring the corresponding absolute wavenumber (cm^{-1}) of different exciting wavelength. As shown in Fig.S3b, the Raman shifts of peaks 1 is 1375 cm^{-1} and 1606 cm^{-1} ex-

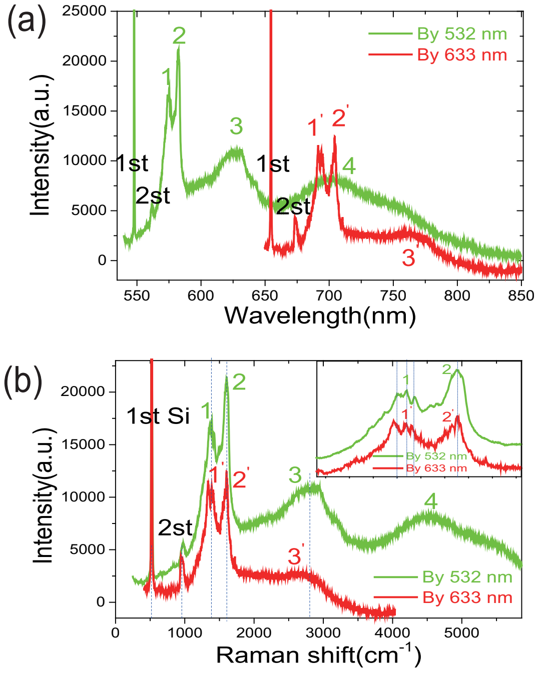

Fig. S 3: (a), The PL spectra of bulk silicon chip with nanowire dimer(NWD) under different exciting laser wavelength of 532 nm and 633 nm, respectively. (b),The Raman spectra of bulk silicon chip under different exciting laser wavelength of 532 nm and 633 nm. Inset is the amplifying of the peaks 1, 2 and 1′, 2′ fine structures which are very similar. Here, 1st and 2st peaks are referred to the first and second order Raman peaks of silicon phonon.

cited by 532 nm wavelength coincide exactly with that of the 1′ , 2′ peaks excited by 633 nm wavelength. Even the fine structure of peak 1 are clearly overlap with that of peak 1′ (Fig.S3b inset). The central position of band 3 is also overlap with 3′ (2795 cm^{-1}). The Raman peaks at 1375 (cm^{-1}), 1606 (cm^{-1}) (1 and 2) are attributed to carbon-carbon covalent bonds of PVP molecules which is juts located in the plasmonic gap, due to the PVP is the envelope of nanowire in our experiment. While the larger Raman shift band of 2795 cm^{-1} is attributed to the $C - H_2$ stretching bonds of PVP molecules.

IV. THEORETICAL SIMULATION ANALYSIS

A. Theoretical simulations methods

The distribution of the electromagnetic field intensity and the surface charge density for nanowire-dimer(NWD) system in this paper were calculated via the finite-difference time-domain (FDTD) method using the Lumerical FDTD Solutions 8.0 software[6]. The NWD configuration here were consisted of double silver nanowires located 2.5 nm above the silicon substrate. The Palik dielectric data for silicon was used. The NWD model for simulation was constructed by the experimental SEM images results. The scattering field of the silver NWD was calculated with a total field and scattering field source acts as a linearly polarized light normally incident on the nanowire systems. The surface charge density is calculated from the Gauss's law. In all calculations, the permittivity of Ag was modeled using the experimental data of Johnson and Christy. The refractive index (RI) of the surfactant PVP layer was set to be 1.5, The simulation domain was finely meshed with a mesh size of 0.25 nm in the gap region, when calculating the LDOS emission patterns, the dipoles sources was launched. A perfectly matched layer boundary condition was introduced to avoid reflection and back scattering of electric field from the preselected boundary. The duration of all simulations was fixed at 500 fs to ensure full electric field convergence.

B. Light Convergence by NWD

Fig.S4 is the calculated Poynting vectors distribution for the single nanowire and the NWD system at 532 nm excitation. For the NWD, the NWD can effectively converge light to the region between the corners of two nanowires.

C. PL enhancement by Single Nanowire

Fig.S5(b) shows the charge distribution of single nanowire on silicon, most of surface charges are confined within a very small volume near the nanogap between nanowire and substrate, resulting in the significant reduction of the total electric dipolar moment,(Fig.S5(e)) is the calculated LDOS and radiation efficiency η. The value of LDOS and radiation efficiency at 680nm are 3.1*107 and 0.017 , respectively. By comparing with the NWD(see the Fig.3(e) in main text), the value of η *LDOS is just one 30th of the NWD, so it hints that this higher order mode always with suppressed radiation [7], or can be called the dark mode.

D. The Calculated Value of Effective Enhancement Factor EF_{eff}

Fig.S6(a) is the calculated electromagnetic distribution profile line at 1 nm above the substrate between two corners of NWD. Fig.S 6(b) is the collection efficiency of the emitters in different position, the 0 nm is the position of A in the main text Fig.3(b), the 40 nm means the position of B. For the emitters in the position A have the minimum excitation E-field enhancement factor 2 and also have the minimum purcell factor 2.4, so we can get the deduced minimum values of the effective enhancement is

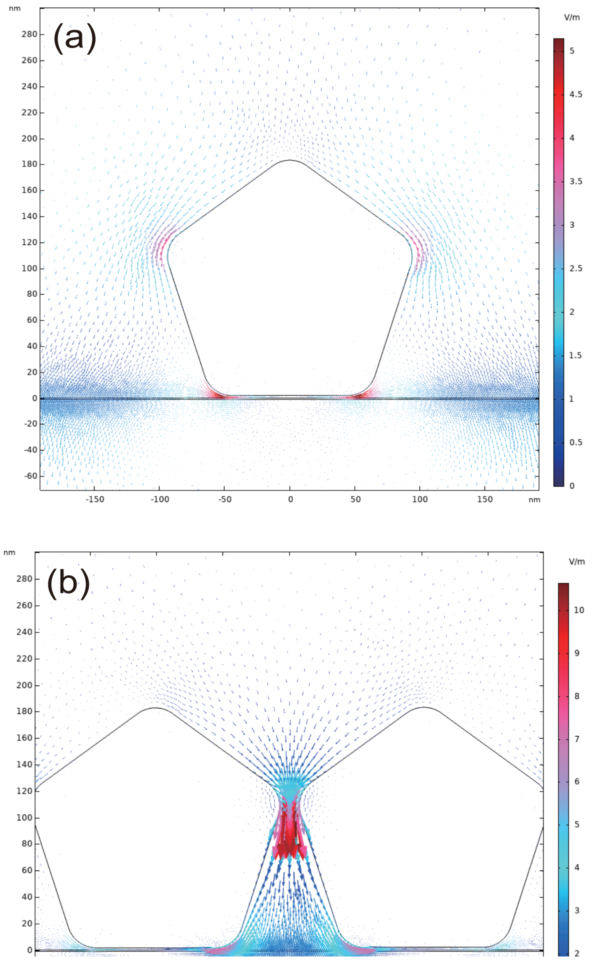

Fig. S 4: (a), The calculated Poynting vectors for a single nanowire; (b), The calculated Poynting vectors for NWD.

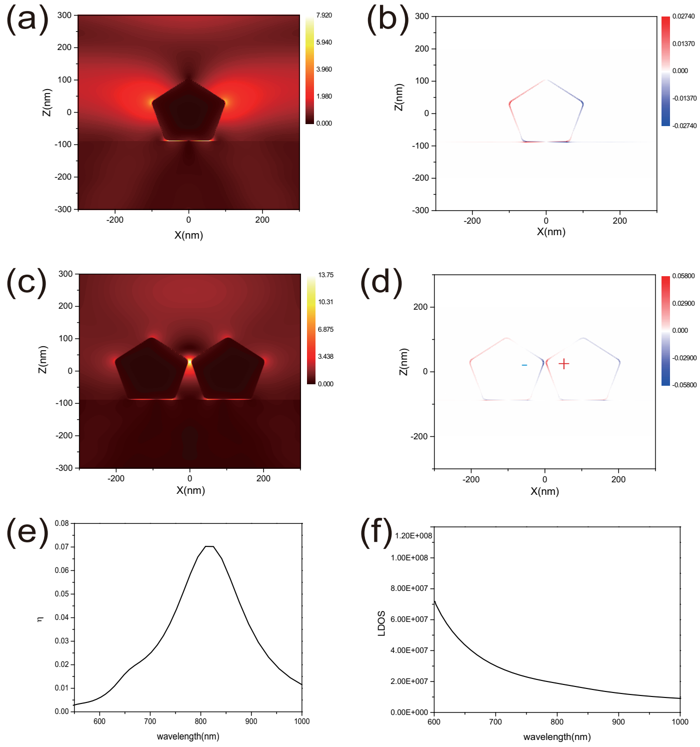

Fig. S 5: (a), (b), The electromagnetic distribution and the surface charge density at 680nm for single nanowire on silicon substrate; (c), (d), The electromagnetic distribution and the surface charge density at 680nm for single nanowire for the N-WD system on silicon substrate. (e), The radiation efficiency η of the single nanowire on silicon system. (f), The calculated LDOS of the single nanowire on silicon system.

50, the maximum effective enhancement up to 2400 happened in the position of 30nm, and both the emitters in this region contribution to the PL enhancement results. Furthermore,we integrated the effective enhancement factor overlap the position of the emitter from d=0nm to $>$ 200nm as shown in as shown in Fig.S7

E. The Calculated Electromagnetic Distribution and the Surface Charge Density for Unparallel NWD

The corresponding electromagnetic distribution and the surface charge density at 680nm is presented in (Fig.S8). which confirms the bonding dipolar mode for the unparallel NWD cases.

F. The Radiation Efficiency η

We calculated the radiation efficiency η of the emitter varies with different distance at the silicon surface as shown in Fig.S9.

V. THE DARK FIELD SCATTERING SPECTRUM OF NWDS

Fig.S10(a) show several representative dark-field(DF) scattering spectrum for NWDs bonding dipole plasmon gap mode. Fig.S10(a) inset are corresponding spatial dark-field images. Fig.S10(b) is the histogram of the plasmonic resonance peaks of the bonding dipole plasmon gap modes. Here the total counted number of NWDs is 100. Most plasmonic resonance peaks of the bonding dipole PGMs are nearby 700 nm.

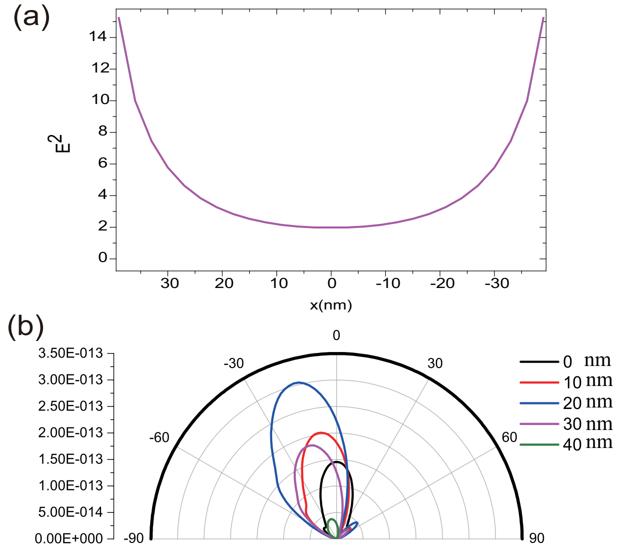

Fig. S 6: (a), The calculated electromagnetic distribution profile line at 1 nm above the substrate between two corners of NWD. Here, the NWD configured were consisted of double silver nanowires located 2.5 nm above the silicon substrate; (b), The collection efficiency of the emitters at different positions(0,10,20,30,40 nm as shown in the main text Fig.3(b)).

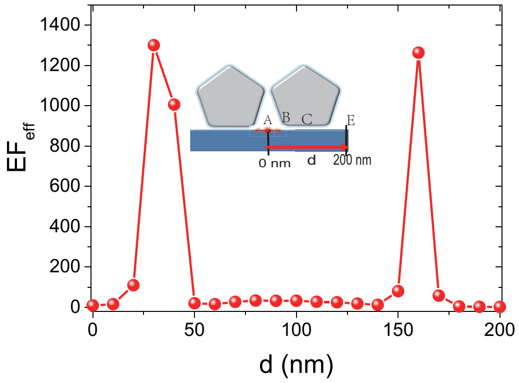

Fig. S 7: The integral effective enhancement factor EF_{eff} of the emitter varies with distance d, from the position A (point A ,d = 0 nm) to position E (d = 200 nm) as shown in the inset.

VI. THE POLARIZATION DEPENDENCE OF PL WITH NWD

Fig.S11 show the PL spectra when the polarization direction of the laser perpendicular and parallel to the long axis direction of the NWD, respectively. It was shown when the polarization of the laser parallel to the nanowire, the PL enhancement is almost impossible to observe. Thats because the surface plasmonic resonance

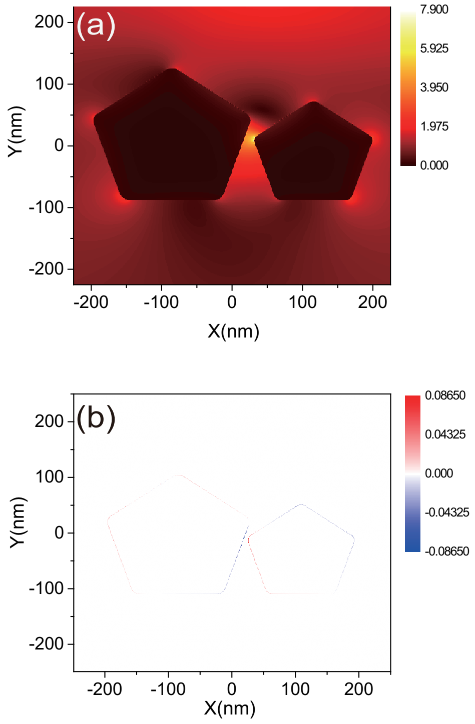

Fig. S 8: a,The electromagnetic distribution and b the surface charge density for the unparallel Ag NWD system with gap of 4 nm on silicon substrate. The exciting laser is set as 685nm which is located at the resonance wavelength region of the surface defect of silicon.

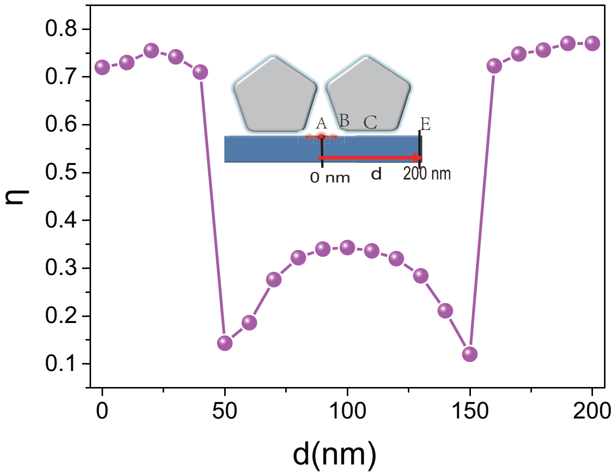

Fig. S 9: The radiation efficiency η of the emitter varies with distance d, from the position A (point A ,d = 0 nm) to position E (d = 200 nm) as shown in the inset.

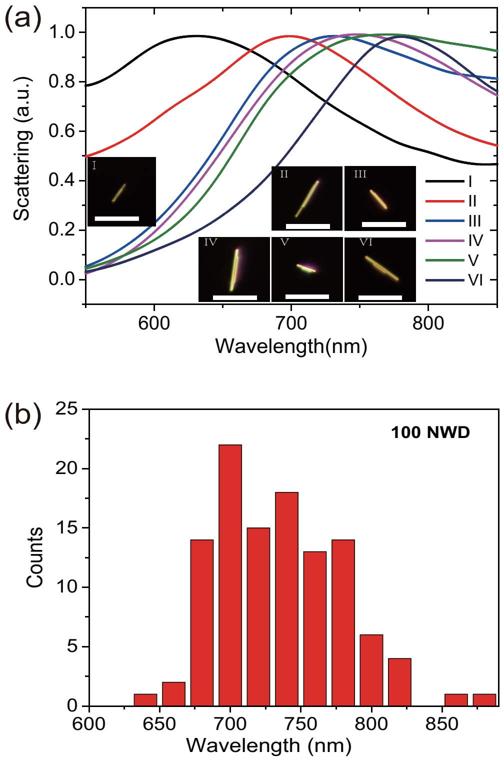

Fig. S 10: (a), The scattering spectrum of six typical NWDs and the corresponding dark-field images inset. All the scale bars are 25 μm; (b), Histogram of the plasmonic resonance peaks of the bonding dipole plasmon gap mode. Here the total counted number of NWDs is 100.

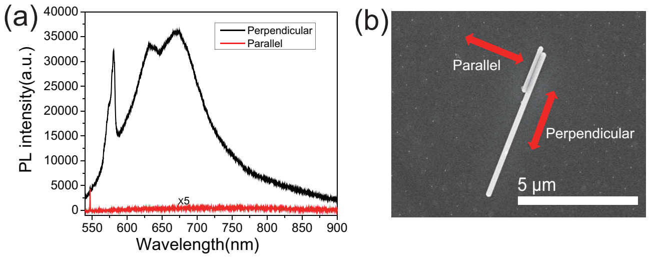

Fig. S 11: (a)The PL spectra when the polarization direction of the laser perpendicular and parallel to the long axis direction of the NWD, respectively; (b), The SEM image of NWD on Si substrate.

mode of the nanogap between the NWD was not excited.

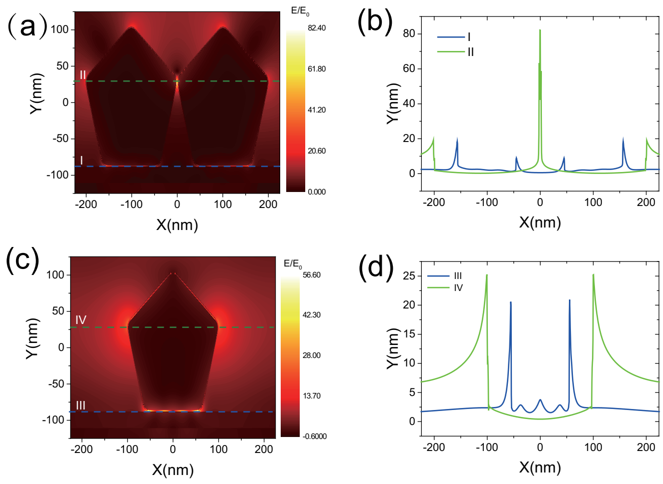

Fig. S 12: (a) and (b) show the electromagnetic field distribution of dimer nanowire and single nanowire at an excitation wavelength of 532 nm. (c)and(d) show the electric field line profiles along the lines shown in (a) and (b), respectively.

VII. THE ELECTROMAGNETIC FIELD DISTRIBUTION OF NANOWIRE DIMER AND SINGLE NANOWIRE

We calculated the field intensity enhancement at the gap between the nanowire dimer/single nanowire and the silicon substrate, and evaluated the field intensity line profile in the gap region as shown in Fig.S12

VIII. THE ELECTROMAGNETIC FIELD DISTRIBUTION OF AG NANOSPHERE DIMER

We calculated the field intensity enhancement at the gap between the nanosphere dimer on silicon substrate, and evaluated the field intensity line profile in the gap region as shown in Fig.S13.

IX. REFERENCES

[1] Li, G. C. et al, Metal-Substrate-Mediated Plasmon Hybridization in a Nanoparticle Dimer for Photoluminescence Line-Width Shrinking and Intensity Enhancement, ACS Nano.,vol.11, pp.3067-3080,2017.
[2] Cai, Y. Y. et al, "Photoluminescence of Gold Nanorods: Purcell Effect Enhanced Emission from Hot Carriers," ACS Nano .,vol.12, pp.976-985,2018.
[3] Viarbitskaya, S. et al, "Tailoring and imaging the plasmonic local density of states in crystalline nanoprisms," Nat. Mater., vol.12, pp.426-432, 2013.

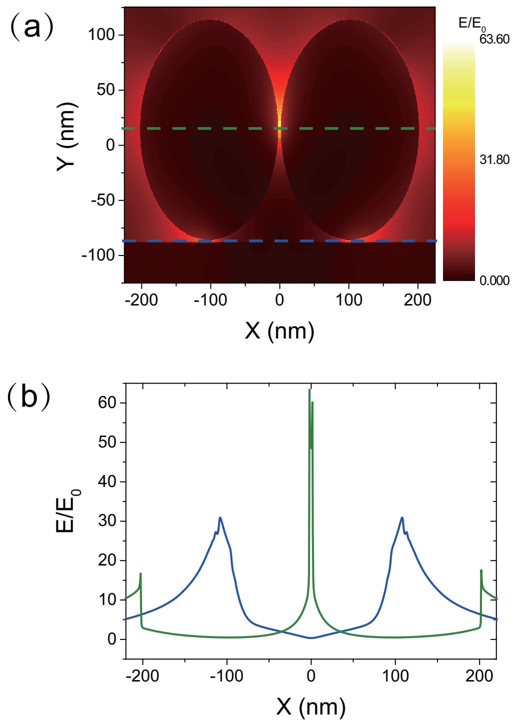

Fig. S 13: (a) show the electromagnetic field distribution of Ag nanosphere dimer on silicon substrate at an excitation wavelength of 532 nm. (b) show the electric field line profiles along the blue and green lines shown in (a), respectively.

[4] Bharadwaj, P. et al, "Optical Antennas," Adv. Opt. Photonics., vol.1, pp.438-483,2009.

[5] Anger, P. et al," Enhancement and quenching of single-molecule fluorescence," Phys. Rev. Lett., vol.96, pp.113002, 2006.

[6] Taflove, A. et al. Hagness, "in Computational Electrodynamics: The Finite-Difference Time-Domain Method," Artech House, Norwood, Mass, USA, 3rd edn, 2005.

[7] Li, G. C. et al, "Hybrid plasmonic gap modes in metal film-coupled dimers and their physical origins revealed by polarization resolved dark field spectroscopy," Nanoscale., vol. 8, pp.7119-7126, 2016.

[8] Cho, C. H. et al, "Silicon coupled with plasmon nanocavity generates bright visible hot-luminescence," Nat. Photonics., vol.7, pp.285-289, 2013.

[9] Aspetti, C. O. et al," Studies of hot photoluminescence in plasmonically coupled silicon via variable energy excitation and temperature-dependent spectroscopy," Nano Lett., vol. 14, pp.5413-5422 ,2014.

[10] Glassner, S. et al," Tuning Electroluminescence from a Plasmonic Cavity-Coupled Silicon Light Source," Nano Lett.,vol. 18, pp.7230-7237, 2018.

[11] Russell, K. J. et al," Hot photoluminescence or Raman scattering," Nat. Photonics., vol.8, pp. 666, 2014.

[12] Aspetti, C. O. et al," Reply to Hot photoluminescence or Raman scattering?," Nat. Photonics., vol.8, pp.667-668 ,2014.
